# Publication Bias in Reports of Animal Stroke Studies Leads to Major Overstatement of Efficacy

**DOI:** 10.1371/journal.pbio.1000344

**Published:** 2010-03-30

**Authors:** Emily S. Sena, H. Bart van der Worp, Philip M. W. Bath, David W. Howells, Malcolm R. Macleod

**Affiliations:** 1 Centre for Clinical Brain Sciences, University of Edinburgh, Edinburgh, United Kingdom; 2 National Stroke Research Institute, Austin Health, University of Melbourne, Melbourne, Victoria, Australia; 3 Department of Medicine, Austin Health, University of Melbourne, Melbourne, Victoria, Australia; 4 Department of Neurology, Rudolf Magnus Institute of Neuroscience, University Medical Center, Utrecht, The Netherlands; 5 Stroke Trials Unit, University of Nottingham, Nottingham, England, United Kingdom; 6 Department of Neurology, NHS Forth Valley, Stirling, Scotland, United Kingdom; London School of Hygiene and Tropical Medicine, United Kingdom

## Abstract

The consolidation of scientific knowledge proceeds through the interpretation and then distillation of data presented in research reports, first in review articles and then in textbooks and undergraduate courses, until truths become accepted as such both amongst “experts” and in the public understanding. Where data are collected but remain unpublished, they cannot contribute to this distillation of knowledge. If these unpublished data differ substantially from published work, conclusions may not reflect adequately the underlying biological effects being described. The existence and any impact of such “publication bias” in the laboratory sciences have not been described. Using the CAMARADES (Collaborative Approach to Meta-analysis and Review of Animal Data in Experimental Studies) database we identified 16 systematic reviews of interventions tested in animal studies of acute ischaemic stroke involving 525 unique publications. Only ten publications (2%) reported no significant effects on infarct volume and only six (1.2%) did not report at least one significant finding. Egger regression and trim-and-fill analysis suggested that publication bias was highly prevalent (present in the literature for 16 and ten interventions, respectively) in animal studies modelling stroke. Trim-and-fill analysis suggested that publication bias might account for around one-third of the efficacy reported in systematic reviews, with reported efficacy falling from 31.3% to 23.8% after adjustment for publication bias. We estimate that a further 214 experiments (in addition to the 1,359 identified through rigorous systematic review; non publication rate 14%) have been conducted but not reported. It is probable that publication bias has an important impact in other animal disease models, and more broadly in the life sciences.

## Abstract 2

Publication bias confounds attempts to use systematic reviews to assess the efficacy of various interventions tested in experiments modelling acute ischaemic stroke, leading to a 30% overstatement of efficacy of interventions tested in animals.

## Author Summary

Publication bias is known to be a major problem in the reporting of clinical trials, but its impact in basic research has not previously been quantified. Here we show that publication bias is prevalent in reports of laboratory-based research in animal models of stroke, such that data from as many as one in seven experiments remain unpublished. The result of this bias is that systematic reviews of the published results of interventions in animal models of stroke overstate their efficacy by around one third. Nonpublication of data raises ethical concerns, first because the animals used have not contributed to the sum of human knowledge, and second because participants in clinical trials may be put at unnecessary risk if efficacy in animals has been overstated. It is unlikely that this publication bias in the basic sciences is restricted to the area we have studied, the preclinical modelling of the efficacy of candidate drugs for stroke. A related article in *PLoS Medicine* (van der Worp et al., doi:10.1371/journal.pmed.1000245) discusses the controversies and possibilities of translating the results of animal experiments into human clinical trials.

## Introduction

Few publications describing natural phenomena are in themselves sufficient to change our understanding of the world, and knowledge advances through the summarising of data in conference presentations, review articles, and books. Traditionally this process has been rather haphazard, with sometimes partisan experts using narrative review articles to emphasise their own particular perspective. Attempts have been made to account for this bias using the technique of systematic review, in which there is prespecification of the biological question being addressed, the methods through which contributing data will be identified, and the criteria that will be used to select which data are included in the analysis [1]. While systematic reviewers often go to some lengths to identify unpublished data sources, both approaches are potentially confounded by the ability to include only available data. If experiments have been conducted but are not available to reviewers, and if the results of these experiments as a group are not the same as results from experiments that were published, then both narrative and systematic reviews, and the resulting expert opinion and public understanding, will be biased. This is the “file drawer problem” [2],[3]: at its most extreme, the 95% of studies that were truly neutral (that is, which reported no significant effects) remain in the files of the investigators, the 5% of experiments that were falsely positive are published, and reviewers conclude—falsely—that the literature represents biological truth.

The consequences of the drawing of erroneous conclusions would be troubling if it involved, for instance, the interpretation of data from clinical trials; indeed, the recognition of a substantial publication bias in this literature has led to the introduction of clinical trial registration systems to ensure that those summarising research findings are at least aware of all relevant clinical trials that have been performed [4]. Publication bias has also been observed in reports of genetic association studies [5] and in ecology and evolution, in which 40% of meta-analyses were confounded by publication bias, and adjusting for publication bias might have altered the conclusions in around one-third of cases [6]. A related group of biases, the citation biases [7], can be addressed through rigorous systematic review, in that an attempt is made to include all relevant publications describing data meeting predefined inclusion or exclusion criteria. However, until recently there has been a paucity of systematic reviews of animal studies [8].

The extent and any impact of publication bias in the experimental sciences are not clear. Timmer and colleagues investigated the process of publication for abstracts submitted to a leading gastrointestinal conference, and suggested both that the most responsibility for nonpublication rested with the authors (76% of unpublished projects were never submitted as a manuscript) and that for basic science studies there was no relationship between the rate of publication and whether the study reported positive, neutral, or negative findings [9]. It has previously not been possible to ascertain the impact of publication bias in animal studies because of the paucity of systematic reviews and meta-analyses, the substantial heterogeneity in the research questions asked in experimental science and in the outcomes reported, and the qualitative rather than quantitative nature of many of those outcomes.

Since 2004 the Collaborative Approach to Meta-Analysis and Review of Animal Data in Experimental Studies (CAMARADES) has curated data collected in the context of systematic reviews of reports of studies describing the efficacy in animals of candidate interventions for stroke [10]–[21]. Here we use that dataset, which includes quantitative data for reported outcomes from individual experiments, to estimate the prevalence and impact of publication bias in laboratory science.

## Results

### Systematic Identification of Previous Reports of Publication Bias in Animal Models of Human Disease

**Figure 1:**
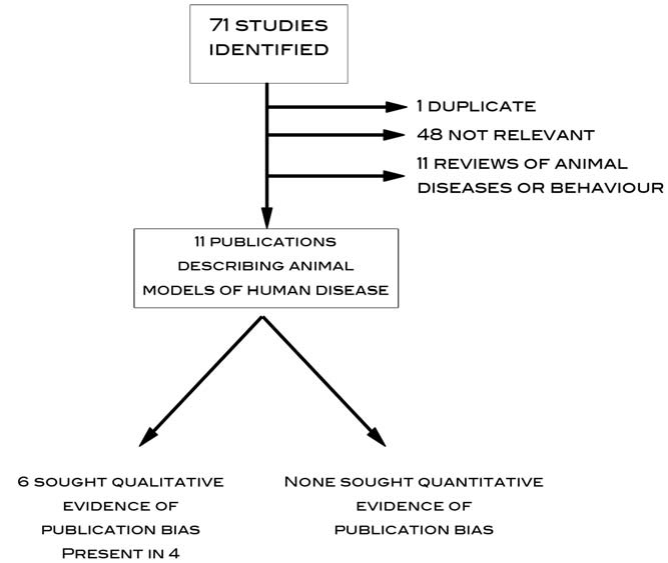
QUOROM chart of fate of 71 publications identified in systematic search for studies reporting the quantitative impact of publication bias in reports of animal experiments modelling human disease.

A search for publications that might have addressed this problem previously identified 71 publications (Text S1). Of these, 11 described meta-analyses of studies reporting the animal modelling of human disease (including three CAMARADES reviews); six reported testing for the presence of publication bias (using funnel plot asymmetry or Egger regression), and publication bias was reported in four (Figure 1). No study gave quantitative estimates of the impact on effect size of publication bias.

### Source Data

As of August 2008 the CAMARADES database contained details from systematic reviews of 16 interventions tested in animal models of stroke. This database comprises data from 525 unique data sources, comprising 514 unique publications (15 contributed data to more than one review) and 11 unpublished communications, describing 1,359 experiments involving 19,956 animals (Table 1). Of these, only ten publications (2%; one publication [22] was represented in reviews of both tirilazad and tPA) described no significant effect on infarct volume, although four reported other statistically significant findings [23]–[26].

**Table 1:** Meta-analyses included in this analysis.

Intervention	No. of Data Sources	No. of Experiments	No. of Animals	Reported Effect Size (95%Cl)
Estrogens [10]	27	99	1,452	26.7% (20.4%–33.0%)
FK506 [12]	27	96	1,596	32.0% (27.8%–36.3%)
Growth factors	70	128	1,750	29.7% (25.9%–33.4%)
Hypothermia [40]	98	222	3,256	43.5% (40.1%–47.0%)
IL1-RA [21]	23	44	784	38.2% (31.2%–45.1%)
Melatonin [13]	12	29	443	42.1% (35.7%–48.5%)
Minocycline	8	25	535	30.9% (24.1%–37.6%)
Nicotinamide [11]	11	57	719	29.2% (23.0%–35.5%)
NOS donors [19]	17	40	483	21.4% (13.7%–29.1%)
NOS inhibitors [41]	52	148	1,998	22.2% (17.1%–27.3%)
NXY-059 [14]	9	29	408	43.8% (34.7%–52.8%)
Piracetam and related compounds [18]	5	14	197	29.6% (16.1%–44.4%)
Stem cells	46	112	1,352	29.6% (23.7%–35.4%)
Tirilazad [16]	18	34	544	31.9% (23.1%–40.7%)
tPA [15]	105	256	4,029	22.5% (19.2%–25.9%)
Other thrombolyics	12	26	410	46.6% (35.7%–57.5%)
**Pooled analysis**	**525** *	**1,359**	**19,956**	**31.3% (29.7%–32.8%)**

*Fifteen data sources were represented in more than one review and are included only once in the pooled analysis.

### Prevalence of Publication Bias

**Figure 2:**
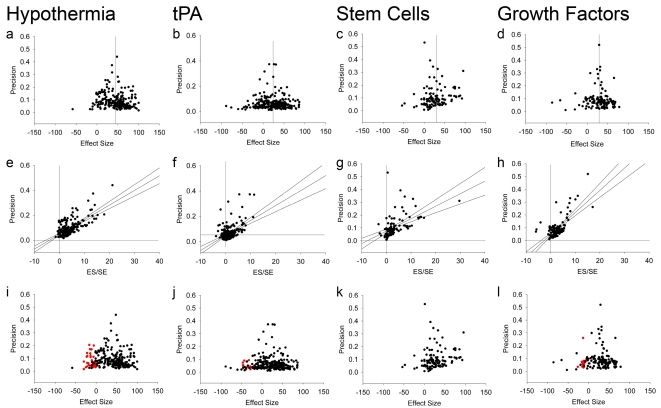
Example funnel plots, Egger regressions, and trim-and-fill plots. Data from meta-analyses of hypothermia (a,e,i), tPA (b,f,j), stem cells (c,g,k), and growth factors (d,h,l). (a–d) Funnel plots showing precision plotted against effect size. In the absence of publication bias the points should resemble an inverted funnel. (e–h) Egger regression showing precision plotted against the standardised effect size. In the absence of publication bias the regression line should pass through the origin. (i–l) Funnel plots showing the data from (a) to (d) in black, and the additional missing studies imputed by trim-and-fill in red.

**Figure 3:**
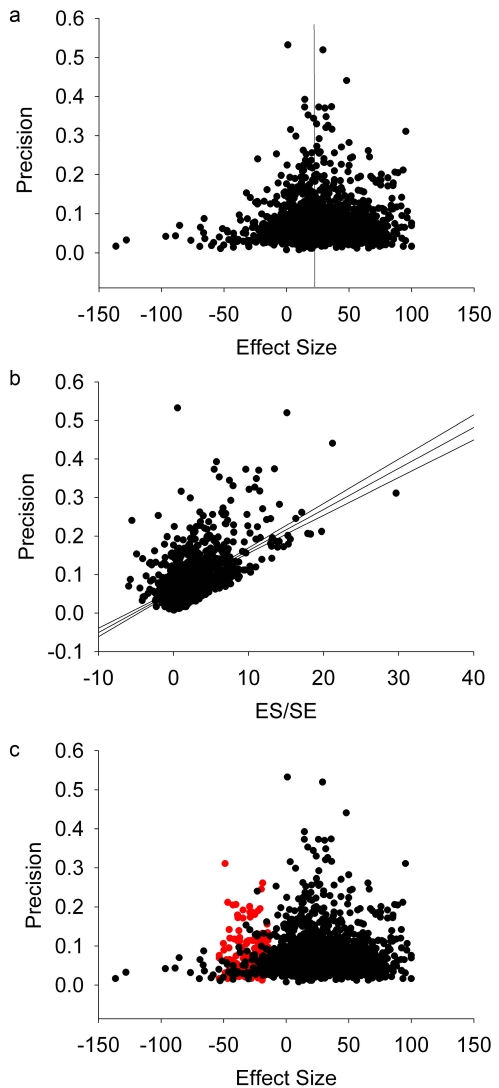
Plots describing the complete dataset. Funnel plot (a), Egger regression (b), and trim-and-fill plots (c). See Figure 1 legend for details.

For individual interventions, visual inspection of funnel plots suggested that only hypothermia and tissue plasminogen activator (tPA) had obvious inverted funnel shapes (examples for hypothermia, tPA, stem cells, and growth factors are shown in Figure 2a–2d). These interventions represented the largest individual datasets (222 experiments involving 3,256 animals and 256 experiments involving 4,029 animals, respectively). However, Egger regression suggested significant asymmetry of all 16 datasets (examples in Figure 2e–2h). In every case the intercept of the regression line was positive, suggesting an excess of imprecise studies reporting large effect sizes over that which would be expected from the overall distribution of the data. Trim-and-fill analysis of individual datasets (examples in Figure 2i–2l) suggested asymmetry affecting ten of 16 interventions, with six interventions without significant asymmetry (minocycline, NXY-059, piracetam, stem cells, tirilazad, and thrombolytics other than tPA). Funnel plotting of the entire dataset showed asymmetry, confirmed by both Egger regression and trim-and-fill (Figure 3; Table 2).

**Table 2:** Prevalence and potential impact of publication bias.

Intervention	Reported Effect Size (95%Cl)	Bias with Egger Regression	Bias with METATRIM	Additional %Studies Considered “Missing”	METATRIM Adjusted Effect Size (95%Cl)	Absolute Overstatement of Efficacy	Relative Overstatement of Efficacy
Estrogens	26.7% (20.4%–33.0%)	+	+	24	11.9% (4.6%–19.2%)^a^	14.8% (8.0%–21.6%)	124.4%
FK506	32.0% (27.8%–36.3%)	+	+	30	21.9% (17.5%–26.3%)^a^	10.1% (5.8%–14.4%)	46.1%
Growth factors	29.7% (25.9%–33.4%)	+	+	14	25.1% (21.2%–28.9%)^a^	4.6% (0.9%–8.3%)	18.3%
Hypothermia	43.5% (40.1%–47.0%)	+	+	20	35.4% (31.7%–39.1%)^a^	8.1% (4.5%–11.6%)	22.9%
IL1-RA	38.2% (31.2%–45.1%)	+	+	36	25.4% (18.4%–32.4%)^a^	12.8% (5.9%–19.7%)	50.4%
Melatonin	42.1% (35.7%–48.5%)	+	+	14	41.0% (34.8%–47.3%)	1.1% (−5.1% to 7.4%)	2.7%
Minocycline	30.9% (24.1%–37.6%)	+	−	0	No adjustment		—
Nicotinamide	29.2% (23.0%–35.5%)	+	+	24	21.8% (14.9%–28.6%)^a^	7.4% (0.8%–13.9%)	33.9%
NOS donors	21.4% (13.7%–29.1%)	+	+	25	14.0% (6.4%–21.6%)^a^	7.4% (−0.1% to 14.9%)	52.9%
NOS inhibitors	22.2% (17.1%–27.3%)	+	+	13	14.7% (8.9%–20.6%)^a^	7.5% (2.0%–13.0%)	51.0%
NXY-059	43.8% (34.7%–52.8%)	+	−	0	No adjustment		—
Piracetam and related compounds	29.6% (16.1%–44.4%)	+	−	0	No adjustment		
Stem cells	29.6% (23.7%–35.4%)	+	−	0	No adjustment		—
Tirilazad	31.9% (23.1%–40.7%)	+	−	0	No adjustment		—
tPA	22.5% (19.2%–25.9%)	+	+	5	19.9% (16.4%–23.3%)	2.6% (−0.7% to 6.0%)	13.1%
Other Thrombolytics	46.6% (35.7%–57.5%)	+	−	0	No adjustment		-
**Pooled analysis**	**31.3% (29.7%–32.8%)**	**+**	**+**	**214** b	**23.8% (22.2%–25.5)** a	**7.5% (5.9%–9.1%)**	**31.1%**

Duval and Tweedie nonparametric trim-and-fill provides an estimate of the number of unpublished studies, and provides an estimate of what the observed efficacy might have been had these studies been available. Where no adjustment is made there are either not enough data to infer the number of missing studies or there is no publication bias.

a *p*<0.05 versus unadjusted estimate of efficacy.

b This (214) is the estimate of missing studies in the pooled analysis of the total dataset rather than the sum of missing studies from the individual drug datasets (205), and suggests that a further nine studies are missing, probably from those reviews where no adjustment was made because the analysis of publication bias was underpowered for smaller reviews.

### Impact of Publication Bias

**Figure 4:**
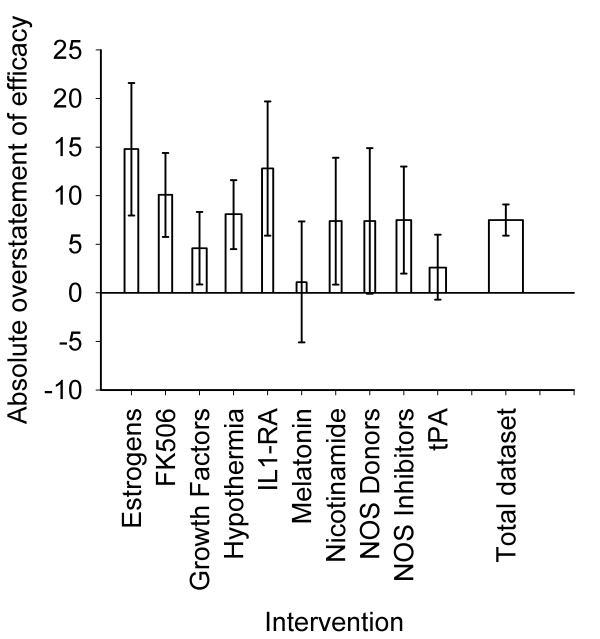
Absolute overstatement of efficacy for the ten interventions identified through trim-and-fill as showing significant publication bias. The vertical error bars represent the 95% confidence intervals of the estimate. The width of each column reflects the log of the number of contributing experiments.

Trim-and-fill imputes the number and most probable results of unpublished experiments to calculate an estimate of what the effect size would be in the absence of publication bias [27] (Figure 2i–2l) The proportion of missing experiments ranged from 5% for tPA to 36% for interleukin 1 receptor antagonist (IL1 RA). Overall, 214 experiments were considered “missing,” or 16% experiments additional to those identified through systematic review (Figure 3c). This is the best estimate for the proportion of studies which were conducted but not reported. Of the ten interventions identified through trim-and-fill as exhibiting significant publication bias, the adjusted effect size was significantly lower than that estimated through conventional meta-analysis in seven. The relative overstatement of efficacy ranged from 2.7% for melatonin to 124.4% for estrogens, and the absolute overstatement of efficacy ranged from 1.1% for melatonin to 14.8% for estrogens, (Figure 4; Table 2). There was no significant association between the number of experiments contributing to a meta-analysis and either the absolute or the relative overstatement of efficacy, and there was no relationship between the precision of individual studies and their methodological quality. Considering all data together, overall efficacy was reduced from 31.2% (95% CI 29.7%–32.8%) to 23.8% (95% CI 22.2%–25.5%; *p*<0.0001) (relative overstatement of efficacy 31.1%; absolute overstatement of efficacy 7.4%).

## Discussion

These data provide to our knowledge the first quantitative estimates of the impact of publication bias in the literature describing animal experiments modelling human disease. Only 2.2% of publications identified in the included reviews did not report any significant findings. While our approach can provide at best only approximations of the magnitude of the problem, our data suggest that effect sizes are inflated by around one-third, and we estimate that around one-sixth of experiments remain unpublished. Many would consider these to be conservative estimates, and indeed a recent systematic review of individual animal data supporting the efficacy of NXY-059 showed that two of four unpublished experiments identified in the course of that review were neutral [28]. The different methods used to assess the presence of publication bias gave somewhat different results, which may reflect the different sensitivities of these approaches. However, it is likely that publication bias is highly prevalent in this literature, and this is likely to bias the conclusions drawn in both narrative and systematic reviews.

The different methods used to ascertain publication bias gave somewhat different results; Egger regression suggested bias for all 16 interventions, whereas trim-and-fill suggested bias for ten of 16 interventions. Importantly, the median number of publications for those interventions in which trim and fill suggested publication bias was higher (27) than those in which publication bias was not found (10.5), suggesting that when publication number is small the trim-and-fill approach may lack statistical power compared with Egger regression.

In discussion of factors that might result in funnel plot asymmetry in animal studies it is important to note that, given their small size and in contrast to clinical trials, variation in study precision relates more to underlying biological variability and to measurement error than to study size. However, there are a number of factors other than publication bias that can cause funnel plot asymmetry [29]:

First, because studies of poorer methodological quality tend to overstate effect sizes [30], lower precision in these studies would lead to funnel plot asymmetry. However, we found no association between study precision and methodological quality in the publications contributing to this analysis.

Second, the effect size may vary according to the size of individual studies. In clinical trials, smaller studies may involve patients at greater risk of an adverse outcome, in whom the intervention is proportionately more effective; or higher doses or more powerful interventions may be used in smaller studies; or smaller studies may focus on particular groups in whom the intervention is more effective. However, none of these features apply to the animal studies examined here.

Third, the studies identified in the individual reviews may not be representative of all studies published. However, the included reviews used detailed search strategies involving multiple electronic databases and conference abstracts; had no language restriction; and where duplicate publication had occurred only one publication was included (see Methods). Selection bias is therefore unlikely.

Finally, if more than one outcome measure was studied, and if effect sizes were consistently higher and precision consistently lower for a particular outcome measure, funnel plot asymmetry would result. However, because this analysis is restricted to studies reporting changes in infarct size, such a problem is unlikely to be an issue here.

In view of the above, it is important to note that, because we have included all data reporting an effect on infarct volume and not just the largest effect size from each publication, we will have included at least some imprecise studies testing ineffective doses (at the lower end of a dose response curve) or at later time points, which could lead to a reversal of funnel plot asymmetry. For this reason, we think that the present study is more likely to underestimate than to overestimate the effect of publication bias.

For meta-analyses of individual interventions, we do not believe that these techniques are sufficiently robust to allow the reliable reporting of a true effect size adjusted for publication bias. This is partly because most meta-analyses are too small to allow reliable reporting, but also because the true effect size may be confounded by many factors, known and unknown, and the empirical usefulness of a precise estimate of efficacy in animals is limited. However, these techniques do allow some estimation both of the presence and of the likely magnitude of publication bias, and reports of meta-analysis of animal studies should include some assessment of the likelihood that publication bias confounds their conclusions, and the possible magnitude of the bias.

These quantitative data raise substantial concerns that publication bias may have a wider impact in attempts to synthesise and summarise data from animal studies and more broadly. It seems highly unlikely that the animal stroke literature is uniquely susceptible to the factors that drive publication bias. First, there is likely to be more enthusiasm amongst scientists, journal editors, and the funders of research for positive than for neutral studies. Second, the vast majority of animal studies do not report sample size calculations and are substantially underpowered. Neutral studies therefore seldom have the statistical power confidently to exclude an effect that would be considered of biological significance, so they are less likely to be published than are similarly underpowered “positive” studies. However, in this context, the positive predictive value of apparently significant results is likely to be substantially lower than the 95% suggested by conventional statistical testing [31]. A further consideration relating to the internal validity of studies is that of study quality. It is now clear that certain aspects of experimental design (particularly randomisation, allocation concealment, and the blinded assessment of outcome) can have a substantial impact on the reported outcome of experiments [14]. While the importance of these issues has been recognised for some years [32], they are rarely reported in contemporary reports of animal experiments [33].

The ethical principles that guide animal studies hold that the number of animals used should be the minimum required to demonstrate the outcome of interest with sufficient precision. For some experiments, this number may be larger than those currently employed. For all experiments involving animals, nonpublication of data means those animals cannot contribute to accumulating knowledge and that research syntheses are likely to overstate biological effects, which may in turn lead to further unnecessary animal experiments testing poorly founded hypotheses. We estimate that for the interventions described here, experiments involving some 3,600 animals have remained unpublished. We consider this practice to be unethical. Others have considered the issue of publication bias in animal stroke studies [34], and have made suggestions for how this might be addressed. Given that a framework regulating animal experimentation already exists in most countries, we suggest that this might be exploited to allow the maintenance of a central register of experiments performed, grouped according to their broad topic, anonymised if required, and referenced in publications arising from that work. Those responsible for preparing conference presentations, review articles, and books would then be much better placed to make a reasonable assessment of the extent to which publication bias may confound their conclusions.

## Methods

We conducted a systematic review for reports of the quantitative impact of publication bias in animal studies by electronic search of PubMed (4 December 2008) with the search term “publication bias”, limited to “animals”. We sought to include publications reporting a quantitative estimate of publication bias in meta-analyses describing the efficacy of interventions in animal models of human disease. Abstracts were independently screened by two investigators (ESS, MRM).

We used data from all meta-analyses (published and unpublished) of interventions tested in animal stroke studies reposited in the database of CAMARADES (an international collaboration established in 2004 to support meta-analyses of animal data for stroke), which had been completed by August 2008. These reviews use a standard methodology including a broad search strategy, inclusion and exclusion criteria, systematic searching of multiple online databases, searching of conference abstracts, and screening of search results by two independent investigators. They perform well against the 12-item checklist for systematic reviews of animal studies (Text S2) proposed by Mignini et al. [35], with a median score of 11 (interquartile range 10.5–11). The CAMARADES data management system includes an analytical package to allow weighted and stratified mean difference meta-analysis; included studies are retained in the database for further analysis, and access to this database is publically available on request.

The database includes details of each individual experiment, including effect size and its standard error. The reviews from which these data are drawn are representative of the literature; they include 11 of a total of 14 meta-analyses of animal studies of stroke which had been published by the end of 2008. Of the five included reviews unpublished at that time (IL1 RA, thrombolytics other than tPA, growth factors, minocycline, stem cells), one has been published and two are under review.

Animal stroke studies report a variety of outcome measures, often measured from the same cohort of animals. To avoid duplication we have restricted the present analysis to reports of effects on infarct size. Where this was determined at multiple time points (for instance using serial MRI), the individual reviews recorded only the last outcome measured. Where a cohort of animals was represented more than once in the database (for instance in studies reporting the effects of tPA and hypothermia in combination), the overall analysis was censored such that each cohort appeared only once. No intervention was the subject of more than one review in the database.

For each experiment, effect size and standard error were extracted. For each intervention, and for all interventions together (with individual experiments being pooled for a global analysis), the prevalence of publication bias was assessed using funnel plotting [36], Egger regression [37], and the Duval and Tweedie nonparametric trim-and-fill approach [27] (enabled in METATRIM, an additional module for STATA).

The basis of funnel plotting and Egger regression is that, all other things being equal, imprecise studies should be as likely to understate efficacy as to overstate it. Where there is a preponderance of imprecise studies overstating efficacy, and all other things being equal, this suggests that imprecise studies understating efficacy are missing from the analysis, as occurs with publication bias. This leads to asymmetry in the funnel plot and to the movement of the Egger regression line *y*-intercept away from the origin.

The basis of trim-and-fill is the identification of the publications contributing most to funnel plot asymmetry, to suppress these from the analysis, and to recalculate the overall estimate of efficacy. Studies contributing most to asymmetry around this new overall estimate are then suppressed, a new estimate calculated, and so the process continues until no further studies are excluded. Then the suppressed studies are replaced, along with matching imputed studies with an effect size calculated by reflection around the recalculated overall estimate and variance equal to that of the study which they are balancing. The number of imputed studies added to the dataset provides an estimate of the number of missing unpublished studies, and meta-analysis of this enlarged dataset provides an approximation of what the true efficacy might be were publication bias not present. We attempted to estimate the extent of publication bias in the animal stroke literature by measuring the relative and absolute differences between the observed estimate of efficacy and the estimated true efficacy.

We tested any relationship between the precision and the methodological quality of individual studies using a ten-item study quality checklist comprising peer-reviewed publication, statement of control of temperature, random allocation to treatment or control, blinded induction of ischemia, blinded assessment of outcome, use of anaesthetic without significant intrinsic neuroprotective activity, appropriate animal model (aged, diabetic, or hypertensive), sample size calculation, compliance with animal welfare regulations, and statement of potential conflict of interests [11]. Despite the potential shortcomings of using aggregate checklist scores rather than assessing the impact of individual study quality items [38], across a range of systematic reviews publications scoring highly on this checklist tend to give lower estimates of treatment effect; while the score has not been formally validated it does have face validity, and has formed the basis for an international consensus statement of Good Laboratory Practice in the modelling of ischaemic stroke [39].

## Supporting Information

Text S1 References identified in search for previous studies of publication bias in animal models.
(0.04 MB DOC)

Text S2 Components of the Mignini checklist.
(0.02 MB DOC)

## Abbreviations

CAMARADES: Collaborative Approach to Meta-analysis and Review of Animal Data in Experimental Studies, tPA, tissue plasminogen activator, IL1-RA, interleukin 1 receptor antagonist

## References

[1] Oxman A. D, Guyatt G. H (1993). The science of reviewing research.. Ann N Y Acad Sci.

[2] Antman E. M, Lau J, Kupelnick B, Mosteller F, Chalmers T. C (1992). A comparison of results of meta-analyses of randomized control trials and recommendations of clinical experts. Treatments for myocardial infarction.. JAMA.

[3] Rosenthal R (1979). The file drawer problem and tolerance for null results.. Psychol Bull.

[4] De Angelis C, Drazen J. M, Frizelle F. A, Haug C, Hoey J (2004). Clinical trial registration: a statement from the International Committee of Medical Journal Editors.. N Engl J Med.

[5] Miettinen O. S (2009). Up from ‘false positives’ in genetic-and other-epidemiology.. Eur J Epidemiol.

[6] Jennions M. D, Moller A. P (2002). Publication bias in ecology and evolution: an empirical assessment using the ‘trim and fill’ method.. Biol Rev Camb Philos Soc.

[7] Greenberg S. A (2009). How citation distortions create unfounded authority: analysis of a citation network.. BMJ.

[8] Pound P, Ebrahim S, Sandercock P, Bracken M. B, Roberts I (2004). Where is the evidence that animal research benefits humans?. BMJ.

[9] Timmer A, Hilsden R. J, Cole J, Hailey D, Sutherland L. R (2002). Publication bias in gastroenterological research - a retrospective cohort study based on abstracts submitted to a scientific meeting.. BMC Med Res Methodol.

[10] Gibson C. L, Gray L. J, Murphy S. P, Bath P. M (2006). Estrogens and experimental ischemic stroke: a systematic review.. J Cereb Blood Flow Metab.

[11] Macleod M. R, O'Collins T, Howells D. W, Donnan G. A (2004). Pooling of animal experimental data reveals influence of study design and publication bias.. Stroke.

[12] Macleod M. R, O'Collins T, Horky L. L, Howells D. W, Donnan G. A (2005). Systematic review and metaanalysis of the efficacy of FK506 in experimental stroke.. J Cereb Blood Flow Metab.

[13] Macleod M. R, O'Collins T, Horky L. L, Howells D. W, Donnan G. A (2005). Systematic review and meta-analysis of the efficacy of melatonin in experimental stroke.. J Pineal Res.

[14] Macleod M. R, van der Worp H. B, Sena E. S, Howells D. W, Dirnagl U (2008). Evidence for the efficacy of NXY-059 in experimental focal cerebral ischaemia is confounded by study quality.. Stroke.

[15] Perel P, Roberts I, Sena E, Wheble P, Briscoe C (2007). Comparison of treatment effects between animal experiments and clinical trials: systematic review.. BMJ.

[16] Sena E, Wheble P, Sandercock P, Macleod M (2007). Systematic Review and Meta-Analysis of the Efficacy of Tirilazad in Experimental Stroke.. Stroke.

[17] van der Worp H. B, Sena E. S, Donnan G. A, Howells D. W, Macleod M. R (2007). Hypothermia in animal models of acute ischaemic stroke: a systematic review and meta-analysis.. Brain.

[18] Wheble P. C, Sena E. S, Macleod M. R (2008). A systematic review and meta-analysis of the efficacy of piracetam and piracetam-like compounds in experimental stroke.. Cerebrovasc Dis.

[19] Willmot M, Gray L, Gibson C, Murphy S, Bath P. M (2005). A systematic review of nitric oxide donors and L-arginine in experimental stroke; effects on infarct size and cerebral blood flow.. Nitric Oxide.

[20] Willmot M, Gibson C, Gray L, Murphy S, Bath P (2005). Nitric oxide synthase inhibitors in experimental ischemic stroke and their effects on infarct size and cerebral blood flow: a systematic review.. Free Radic Biol Med.

[21] Banwell V, Sena E. S, Macleod M. R (2009). Systematic review and stratified meta-analysis of the efficacy of interleukin-1 receptor antagonist in animal models of stroke.. J Stroke Cerebrovasc Dis.

[22] Orozco J, Mendel R. C, Hahn M. R, Guthkelch A. N, Carter L. P (1995). Influence of a ‘brain protector’ drug 21-amino steroid on the effects of experimental embolic stroke treated by thrombolysis.. Neurol Res.

[23] Prado R, Watson B. D, Zhao W, Yao H, Busto R (1996). L-arginine does not improve cortical perfusion or histopathological outcome in spontaneously hypertensive rats subjected to distal middle cerebral artery photothrombotic occlusion.. J Cereb Blood Flow Metab.

[24] Roberts T. P, Vexler Z. S, Derugin N, Kozniewska E, Kucharczyk J (1995). Evaluation of recombinant human basic fibroblast growth factor (rhbFGF) as a cerebroprotective agent using high speed MR imaging.. Brain Res.

[25] Dawson D. A, Kusumoto K, Graham D. I, McCulloch J, Macrae I. M (1992). Inhibition of nitric oxide synthesis does not reduce infarct volume in a rat model of focal cerebral ischaemia.. Neurosci Lett.

[26] Oprica M, Van Dam A. M, Lundkvist J, Iverfeldt K, Winblad B (2004). Effects of chronic overexpression of interleukin-1 receptor antagonist in a model of permanent focal cerebral ischemia in mouse.. Acta Neuropathol.

[27] Duval S, Tweedie R (2000). A nonparametric “trim and fill” method of accounting for publication bias in meta-analysis.. Jounal of the American Statistical Association.

[28] Bath P. M, Gray L. J, Bath A. J, Buchan A, Miyata T (2009). Effects of NXY-059 in experimental stroke: an individual animal meta-analysis.. Br J Pharmacol.

[29] Lau J, Ioannidis J. P. A, Terrin N, Schmid C. H, Olkin I (2006). The case of the misleading funnel plot.. BMJ.

[30] Egger M, Davey S. G, Schneider M, Minder C (1997). Bias in meta-analysis detected by a simple, graphical test.. BMJ.

[31] Ioannidis J. P (2005). Why most published research findings are false.. PLoS Med.

[32] Fisher R. A (1936). The Design of Experiments..

[33] Sena E, van der Worp H. B, Howells D, Macleod M (2007). How can we improve the pre-clinical development of drugs for stroke?. Trends Neurosci.

[34] Shimin L (2009). Dealing with publication bias in translational stroke research.. J Exp Stroke Transl Med.

[35] Mignini L. E, Khan K. S (2006). Methodological quality of systematic reviews of animal studies: a survey of reviews of basic research.. BMC Med Res Methodol.

[36] Light R. J, Pillemer D. B (1984). Summing Up: The Science of Reviewing Research..

[37] Egger M, Davey S. G, Schneider M, Minder C (1997). Bias in meta-analysis detected by a simple, graphical test.. BMJ.

[38] Juni P, Altman D. G, Egger M (2001). Systematic reviews in health care: Assessing the quality of controlled clinical trials.. BMJ.

[39] Macleod M. R, Fisher M, O'Collins V, Sena E. S, Dirnagl U (2009). Good laboratory practice: preventing introduction of bias at the bench.. Stroke.

[40] van der Worp H. B, Sena E. S, Donnan G. A, Howells D. W, Macleod M. R (2007). Hypothermia in animal models of acute ischaemic stroke: a systematic review and meta-analysis.. Brain.

[41] Willmot M, Gibson C, Gray L, Murphy S, Bath P (2005). Nitric oxide synthase inhibitors in experimental ischemic stroke and their effects on infarct size and cerebral blood flow: a systematic review.. Free Radic Biol Med.

