# Supplementary material for: Publication Bias in Reports of Animal Stroke Studies Leads to Major Overstatement of Efficacy
Source: PLoS Biol. 2010 Mar 30;8(3):e1000344. doi: 10.1371/journal.pbio.1000344 (PMC2846857; doi:10.1371/journal.pbio.1000344)
Supplement: Text S1 — References identified in search for previous studies of publication bias in animal models. (0.04 MB DOC) [file pbio.1000344.s001.doc]

Text S1

Appendix 1: References identified in search for previous studies of publication bias in animal models

1. VIjayalaxmi, Prihoda TJ (2008) Genetic damage in mammalian somatic cells exposed to radiofrequency radiation: a meta-analysis of data from 63 publications (1990-2005). Radiat Res 169: 561-574.

2. Prego R, Santos-Echeandia J, Cobelo-Garcia A (2008) Letter to the editor re: Villares et al., 2007; on the impact of the Prestige oil spill on the levels of vanadium and other trace elements along the coast of Galicia (NW Iberian Peninsula). Sci Total Environ 399: 216-218.

3. Tenhunen JJ (2008) Bull's eye missed by the magic bullet: preclinical investigations, publication bias, and promising new interventions. Crit Care Med 36: 1361-1363.

4. Duffield TF, Rabiee AR, Lean IJ (2008) A meta-analysis of the impact of monensin in lactating dairy cattle. Part 2. Production effects. J Dairy Sci 91: 1347-1360.

5. Duffield TF, Rabiee AR, Lean IJ (2008) A meta-analysis of the impact of monensin in lactating dairy cattle. Part 1. Metabolic effects. J Dairy Sci 91: 1334-1346.

6. Neitzke U, Harder T, Schellong K, Melchior K, Ziska T, et al. (2008) Intrauterine growth restriction in a rodent model and developmental programming of the metabolic syndrome: a critical appraisal of the experimental evidence. Placenta 29: 246-254.

7. Navas-Acien A, Bleys J, Guallar E (2008) Selenium intake and cardiovascular risk: what is new? Curr Opin Lipidol 19: 43-49.

8. Jaffrezic F, de Koning DJ, Boettcher PJ, Bonnet A, Buitenhuis B, et al. (2007) Analysis of the real EADGENE data set: comparison of methods and guidelines for data normalisation and selection of differentially expressed genes (open access publication). Genet Sel Evol 39: 633-650.

9. Wheble PC, Sena ES, Macleod MR (2008) A systematic review and meta-analysis of the efficacy of piracetam and piracetam-like compounds in experimental stroke. Cerebrovasc Dis 25: 5-11.

10. Knapczyk FN, Conner JK (2007) Estimates of the average strength of natural selection are not inflated by sampling error or publication bias. Am Nat 170: 501-508.

11. Rollin BE (2007) An ethicist's commentary on avoiding impropriety in corporate sponsorship of conferences and journals. Can Vet J 48: 792.

12. Sena E, van der Worp HB, Howells D, Macleod M (2007) How can we improve the pre-clinical development of drugs for stroke? Trends Neurosci 30: 433-439.

13. Qin LQ, Xu JY, Wang PY, Tong J, Hoshi K (2007) Milk consumption is a risk factor for prostate cancer in Western countries: evidence from cohort studies. Asia Pac J Clin Nutr 16: 467-476.

14. Ferraro G (2007) Comments on Carpenter, A., 2007 "The Bonn Agreement Aerial Surveillance Programme: Trends in North Sea Oil Pollution 1986-2004", Marine Pollution Bulletin, vol. 54, 2007, pp. 149-163. Mar Pollut Bull 54: 1072-1074.

15. Nespolo RF, Franco M (2007) Whole-animal metabolic rate is a repeatable trait: a meta-analysis. J Exp Biol 210: 2000-2005.

16. Fountoulakis KN, Vieta E, Bouras C, Notaridis G, Giannakopoulos P, et al. (2008) A systematic review of existing data on long-term lithium therapy: neuroprotective or neurotoxic? Int J Neuropsychopharmacol 11: 269-287.

17. De Ponti F, Tonini M (2007) Publication of a negative trial without disclosing the drug. Aliment Pharmacol Ther 25: 1247-1248.

18. Lawson Handley LJ, Perrin N (2007) Advances in our understanding of mammalian sex-biased dispersal. Mol Ecol 16: 1559-1578.

19. Benatar D (2007) Unscientific ethics: science and selective ethics. Hastings Cent Rep 37: 30-32.

20. Fontana L, Klein S (2007) Aging, adiposity, and calorie restriction. JAMA 297: 986-994.

21. Benatar M (2007) Lost in translation: treatment trials in the SOD1 mouse and in human ALS. Neurobiol Dis 26: 1-13.

22. Toh S, Hernandez-Diaz S (2007) Statins and fracture risk. A systematic review. Pharmacoepidemiol Drug Saf 16: 627-640.

23. Juutilainen J, Kumlin T, Naarala J (2006) Do extremely low frequency magnetic fields enhance the effects of environmental carcinogens? A meta-analysis of experimental studies. Int J Radiat Biol 82: 1-12.

24. Mignini LE, Khan KS (2006) Methodological quality of systematic reviews of animal studies: a survey of reviews of basic research. BMC Med Res Methodol 6:10.: 10.

25. Dirnagl U (2006) Bench to bedside: the quest for quality in experimental stroke research. J Cereb Blood Flow Metab 26: 1465-1478.

26. O'Brien SJ, Gillespie IA, Sivanesan MA, Elson R, Hughes C, et al. (2006) Publication bias in foodborne outbreaks of infectious intestinal disease and its implications for evidence-based food policy. England and Wales 1992-2003. Epidemiol Infect 134: 667-674.

27. Ballantyne B (2005) The occupational toxicologist: professionalism, morality and ethical standards in the context of legal and non-litigation issues. J Appl Toxicol 25: 496-513.

28. Gueorguieva RV (2005) Comments about Joint Modeling of Cluster Size and Binary and Continuous Subunit-Specific Outcomes. Biometrics 61: 862-866.

29. Macleod MR, O'Collins T, Horky LL, Howells DW, Donnan GA (2005) Systematic review and metaanalysis of the efficacy of FK506 in experimental stroke. J Cereb Blood Flow Metab 25: 713-721.

30. Johansson EE (2004) [Story and evidence about pain and gender. "The Princess on the Pea"--a myth about femininity penetrating to sciences?]. Lakartidningen 101: 3774, 3776, 3778-3774, 3776, 3779.

31. Bacchetti P (2005) Uncertainty due to model choice in variant Creutzfeldt-Jakob disease projections. Stat Med 24: 83-93.

32. Munafo MR, Flint J (2004) Meta-analysis of genetic association studies. Trends Genet 20: 439-444.

33. Both C, Artemyev AV, Blaauw B, Cowie RJ, Dekhuijzen AJ, et al. (2004) Large-scale geographical variation confirms that climate change causes birds to lay earlier. Proc Biol Sci 271: 1657-1662.

34. Kaplan BJ, Andrus GM, Parish WW (2004) Germane facts about germanium sesquioxide: II. Scientific error and misrepresentation. J Altern Complement Med 10: 345-348.

35. Sanchez J, Dohoo I, Carrier J, DesCoteaux L (2004) A meta-analysis of the milk-production response after anthelmintic treatment in naturally infected adult dairy cows. Prev Vet Med 63: 237-256.

36. Macleod MR, O'Collins T, Howells DW, Donnan GA (2004) Pooling of animal experimental data reveals influence of study design and publication bias. Stroke 35: 1203-1208.

37. Zhou Y, Cheng YS (2004) Dosimetry of metal tritide particles as evaluated by the ICRP 66 model and a biokinetic model from laboratory rats. Health Phys 86: 155-160.

38. Huff J (2003) IARC and the DEHP quagmire. Int J Occup Environ Health 9: 402-404.

39. Ernst E (2003) The benefits of Arnica: 16 case reports. Homeopathy 92: 217-219.

40. Timmermans S (2003) A black technician and blue babies. Soc Stud Sci 33: 197-229.

41. Kelly JJ, Auer RN (2003) Mefenamate, an agent that fails to attenuate experimental cerebral infarction. Can J Neurol Sci 30: 259-262.

42. Dirx MJ, Zeegers MP, Dagnelie PC, van den BT, van den Brandt PA (2003) Energy restriction and the risk of spontaneous mammary tumors in mice: a meta-analysis. Int J Cancer %20;106: 766-770.

43. Sinclair BJ, Addo-Bediako A, Chown SL (2003) Climatic variability and the evolution of insect freeze tolerance. Biol Rev Camb Philos Soc 78: 181-195.

44. Mirer FE (2003) Comment from the union participant in the IARC Working Group that downgraded DEHP. Int J Occup Environ Health 9: 85-87.

45. Melnick RL (2003) Suppression of crucial information in the IARC evaluation of DEHP. Int J Occup Environ Health 9: 84-85.

46. Hoyt CS (2003) Interesting idea-prove it! Br J Ophthalmol 87: 1-2.

47. Sass J (2002) Lead IARC towards compliance with WHO/IARC Declaration of Interests (DOI) policy. Int J Occup Environ Health 8: 277-278.

48. Huff J (2002) IARC monographs, industry influence, and upgrading, downgrading, and under-grading chemicals: a personal point of view. International Agency for Research on Cancer. Int J Occup Environ Health 8: 249-270.

49. Persson A, Welin S (2002) [Difficult meeting between science and market forces]. Lakartidningen 99: 2022-2026.

50. Jennions MD, Moller AP (2002) Publication bias in ecology and evolution: an empirical assessment using the 'trim and fill' method. Biol Rev Camb Philos Soc 77: 211-222.

51. Lee PN (2002) Environmental tobacco smoke and cancer of sites other than the lung in adult non-smokers. Food Chem Toxicol 40: 747-766.

52. Hasselquist D, Kempenaers B (2002) Parental care and adaptive brood sex ratio manipulation in birds. Philos Trans R Soc Lond B Biol Sci 357: 363-372.

53. Samuels A (1999) The toxicity/safety of processed free glutamic acid (MSG): a study in suppression of information. Account Res 6: 259-310.

54. Birke L, Michael M (1998) The heart of the matter: animal bodies, ethics, and species boundaries. Soc Anim 6: 245-261.

55. Kruse CR (1998) Who said that? Status presentation in media accounts of the animal experimentation debate. Soc Anim 6: 235-243.

56. Priyadarshi A, Khuder SA, Schaub EA, Priyadarshi SS (2001) Environmental risk factors and Parkinson's disease: a metaanalysis. Environ Res 86: 122-127.

57. Fleischauer AT, Arab L (2001) Garlic and cancer: a critical review of the epidemiologic literature. J Nutr 131: 1032S-1040S.

58. Eloubeidi MA, Wade SB, Provenzale D (2001) Factors associated with acceptance and full publication of GI endoscopic research originally published in abstract form. Gastrointest Endosc 53: 275-282.

59. Menger MD, Vollmar B (2000) [Publishing of negative results in experimental research]. Dtsch Med Wochenschr 125: 1028.

60. Bonate PL, Howard D (2000) Prospective allometric scaling: does the emperor have clothes? J Clin Pharmacol 40: 665-670.

61. Poulin R (2000) Manipulation of host behaviour by parasites: a weakening paradigm? Proc Biol Sci 267: 787-792.

62. Bonate PL, Howard D (2000) Prospective allometric scaling: does the emperor have clothes? J Clin Pharmacol 40: 335-340.

63. Vollestad LA, Hindar K, Moller AP (1999) A meta-analysis of fluctuating asymmetry in relation to heterozygosity. Heredity 83: 206-218.

64. Ernst E, Resch KL (1999) Reviewer bias against the unconventional? A randomized double-blind study of peer review. Complement Ther Med 7: 19-23.

65. Jacobson RM (1999) Promises and pitfalls of meta-analysis in vaccine research. Vaccine 17: 1628-1634.

66. Misakian AL, Bero LA (1998) Publication bias and research on passive smoking: comparison of published and unpublished studies. JAMA 280: 250-253.

67. La Vecchia C, Tavani A (1995) Epidemiological evidence on hair dyes and the risk of cancer in humans. Eur J Cancer Prev 4: 31-43.

68. Evans MA, Bonett DG (1994) Bias reduction for multiple-recapture estimators of closed population size. Biometrics 50: 388-395.

69. Staessen JA, Lauwerys RR, Bulpitt CJ, Fagard R, Lijnen P, et al. (1994) Is a positive association between lead exposure and blood pressure supported by animal experiments? Curr Opin Nephrol Hypertens 3: 257-263.

70. McGregor DB, Pangrekar J, Rosenkranz HS, Klopman G (1994) A reexamination of the low prevalence of carcinogens in an early carcinogen screen. Regul Toxicol Pharmacol 19: 97-105.

71. Hayakawa K, Hardy RR, Herzenberg LA (1986) Peritoneal Ly-1 B cells: genetic control, autoantibody production, increased lambda light chain expression. Eur J Immunol 16: 450-456.
