# Supplementary material for: Publication Bias in Reports of Animal Stroke Studies Leads to Major Overstatement of Efficacy
Source: PLoS Biol. 2010 Mar 30;8(3):e1000344. doi: 10.1371/journal.pbio.1000344 (PMC2846857; doi:10.1371/journal.pbio.1000344)
Supplement: Text S2 — Components of the Mignini checklist. (0.02 MB DOC) [file pbio.1000344.s002.doc]

Text S2

Appendix 2: Components of the Mignini checklist

Framing the question:

1. Question specified
2. Narrow focus of the question
3. Explicit testable hypothesis

Literature search:

1. Search description
2. Use of multiple databases
3. Use of reference list
4. Search without language restriction
5. Assessment for risk of missing studies

Methods of reviews:

1. Study quality assessment
2. Tabulation of findings
3. Assessment for heterogeneity
4. Meta-analysis
